# Measurement of absorption and reduced scattering coefficients in Asian human epidermis, dermis, and subcutaneous fat tissues in the 400- to 1100-nm wavelength range for optical penetration depth and energy deposition analysis

**DOI:** 10.1117/1.JBO.25.4.045002

**Published:** 2020-04-30

**Authors:** Yu Shimojo, Takahiro Nishimura, Hisanao Hazama, Toshiyuki Ozawa, Kunio Awazu

**Affiliations:** a Osaka University, Graduate School of Engineering, Suita, Japan; b Osaka City University, Graduate School of Medicine, Department of Dermatology, Osaka, Japan; c Osaka University, Graduate School of Frontier Biosciences, Suita, Japan; d Osaka University, Global Center for Medical Engineering and Informatics, Suita, Japan

**Keywords:** Asian skin tissues, absorption coefficient, reduced scattering coefficient, epidermis, dermis, subcutaneous fat

## Abstract

**Significance:** In laser therapy and diagnosis of skin diseases, the irradiated light distribution, which is determined by the absorption coefficient $\mu_{a}$ and reduced scattering coefficient $\mu_{s}^{\prime }$ of the epidermis, dermis, and subcutaneous fat, affects the treatment outcome and diagnosis accuracy. Although values for $\mu_{a}$ and $\mu_{s}^{\prime }$ have been reported, detailed analysis for Asian skin tissues is still lacking.

**Aim:** We present $\mu_{a}$ and $\mu_{s}^{\prime }$ measurements of Asian skin tissues in the 400- to 1100-nm wavelength range for evaluating optical penetration depth and energy deposition.

**Approach:** The measurements with Asian human skin samples are performed employing a double integrating sphere spectrometric system and an inverse Monte Carlo technique. Using the measured parameters, the optical penetration depth and energy deposition are quantitatively analyzed.

**Results:** The $\mu_{a}$ of the epidermis layer varies among different ethnic groups, while the $\mu_{a}$ of the other layers and the $\mu_{s}^{\prime }$ of all of the layers exhibit almost no differences. The analysis reveals that the optical penetration depth and the energy deposition affect the photodynamic therapy treatment depth and the heat production in skin tissue, respectively.

**Conclusions:** The experimentally measured values of $\mu_{a}$ and $\mu_{s}^{\prime }$ for Asian skin tissues are presented, and the light behavior in Asian skin tissues is analyzed using a layered tissue model.

## 1 Introduction

In laser therapy and diagnosis of skin diseases, the light distribution in tissues provides fundamental information to estimate treatment outcomes and improve diagnosis accuracy.^1^ The irradiated light for therapy and diagnosis is scattered and absorbed while propagating through skin tissue. The absorbed light is translated into thermal,^2^ chemical,^3^ mechanical,^4^ and biological^5^ effects. The light–tissue interactions, which are caused by the light distributions, result in the therapeutic effects and the diagnostic responses reflecting the skin condition. A computational approach is available for analyzing light distributions in skin tissue based on a Monte Carlo technique and the use of numerical parameters such as tissue optical properties and tissue geometries.^6^^,^^7^ The accuracy of the parameters affects the analysis results. Skin tissue structure is modeled as three layers comprising the epidermis, dermis, and subcutaneous fat.^8^^,^^9^ The optical properties of skin tissue, especially of the epidermis, dermis, and subcutaneous fat, are key parameters for obtaining adequate light distributions.

The optical properties comprising the absorption coefficient $\mu_{a}$ and reduced scattering coefficient $\mu_{s}^{\prime }$ of human epidermis, dermis, and subcutaneous fat have been investigated for the high-accuracy calculation of light distributions in skin tissue.^10^^–^^13^ The $\mu_{a}$ and $\mu_{s}^{\prime }$ of skin tissue differ among different ethnic groups. Appropriate irradiation protocols for laser therapy and diagnosis require taking the differences in skin optical properties into account. Several papers have reported the $\mu_{a}$ and $\mu_{s}^{\prime }$ of each layer of skin tissue obtained from *in vitro* measurements;^10^^,^^11^ however, all of these works have measured only Caucasian and African skin tissues. The validity of estimating the $\mu_{a}$ value of each layer in Asian skin tissues from the concentrations of chromophores, such as blood, water, and melanin,^14^ has not yet been verified in detail. So far, *in vivo* measurements have been proposed.^12^^,^^13^ However, the $\mu_{a}$, $\mu_{s}^{\prime }$, and structural information for each layer cannot be easily detected separately because a large number of variables are involved when solving the inverse problems.^15^^,^^16^ The construction of a layered skin model with the measured $\mu_{a}$ and $\mu_{s}^{\prime }$ values allows for the light distributions in Asian skin tissue to be determined with higher accuracy.

This paper presents $\mu_{a}$ and $\mu_{s}^{\prime }$ measurements of Asian epidermis, dermis, and subcutaneous fat tissues in the visible and near-infrared wavelength range. For the measurements, a double integrating sphere spectrometric system and an inverse Monte Carlo (iMC) technique were employed.^17^ The use of this spectrometric system allows for the simultaneous measurement of the diffuse reflectance $R_{d}$ and total transmittance $T_{t}$ and reduces the sample degradation during measurements. Comparing the measured $R_{d}$ and $T_{t}$ with simulated $R_{d}$ and $T_{t}$ using the iMC technique, the $\mu_{a}$ and $\mu_{s}^{\prime }$ of Asian epidermis, dermis, and subcutaneous fat can be obtained. To confirm the validity of the measured $\mu_{a}$ and $\mu_{s}^{\prime }$ of each layer in Asian skin tissues, the $\mu_{a}$ and $\mu_{s}^{\prime }$ values were compared with the reported values for Caucasian and African tissues. Using a layered skin model with these parameters combined with a Monte Carlo simulation, the optical penetration depth and energy deposition during laser irradiation were quantitatively analyzed to investigate the ethnic differences in light effects on skin tissue. Laser and light-based skin treatments deliver light to the target layer of skin tissue with caution to avoid damage to the surrounding tissue layers.^18^^–^^20^ The analysis with a layered skin model provides dermatologists and plastic surgeons with a better comprehension of the light behavior in each layer of skin tissue and how changes in the skin types can cause differences in the light distributions.

## 2 Materials and Methods

### 2.1 Sample Preparation

Freshly discarded specimens of Japanese human skin tissue were obtained from the surgeries at the Department of Dermatology, Osaka City University, immersed in saline solution, and then delivered to Osaka University. A study protocol approved by the Research Ethics Committee of Osaka University (approval number: R 29) and the Ethical Committee of Osaka City University Graduate School of Medicine (approval number: 4252) was followed, and an informed consent form was signed by each participating patient before surgery. Skin excisions from the face, abdomen, thigh, axilla, clavicle, and ear of the adult patients were used. The specimens were stored at low temperature (4°C) until sectioned into the epidermis, dermis, and subcutaneous fat for spectroscopic measurements. The number of sectioned specimens were 21, 20, and 15, respectively. In total, 15 samples of epidermis, 20 samples of dermis, and 15 samples of subcutaneous fat were successfully separated and subsequently measured and analyzed. The time taken from the sample preparation to the measurement did not exceed 50 h for epidermis and dermis and 12 h for subcutaneous fat. The subcutaneous fat was cut using surgical scissors. The epidermis and dermis were then separated with surgical tweezers using a split skin technique.^21^ The human skin specimens were incubated in a phosphate saline buffer (PBS) (163-25265, FUJIFILM Wako Pure Chemical Corporation, Japan) containing 2 mM ethylenediaminetetraacetic acid (06894-14, Nacalai Tesque, Japan) and 1 mM phenylmethylsulfonyl fluoride (160-12183, FUJIFILM Wako Pure Chemical Corporation) for 48 h at 4°C with a daily exchange of the solution. The thickness of each section was measured at three points using a high-precision digital micrometer (MDE-25MX, Mitutoyo, Japan) with a precision of ±1  μm and averaged. The thickness of the epidermis, dermis, and subcutaneous fat sections varied from 0.09 to 0.32 mm, 1.03 to 2.10 mm, and 1.21 to 1.90 mm, respectively. The lateral size of the sectioned tissues was around $20\times 20\;{\mathrm{mm}}^{2}$. The sectioned specimens were sandwiched between glass slides (S1112, Matsunami Glass Ind., Japan) without (or with minimal) compression, and the thickness was fixed using spacers. The samples were sealed up with scotch tape to prevent desiccation during the measurements.

### 2.2 Double Integrating Sphere Spectrometer

The optical parameters comprising the diffuse reflectance $R_{d}$ and total transmittance $T_{t}$ were obtained using a double integrating sphere spectrometric system as reported previously.^17^ In the system, a xenon lamp (L2273 and C8849, Hamamatsu Photonics, Japan) was used as the white light source. The light was focused into 1 mm spots on the samples, which were mounted between the 100-mm outer diameter reflectance and transmittance spheres (CSTM-3P-GPS-033SL, Labsphere). The integrating spheres were made of Spectralon, which is a solid thermoplastic that exhibits the highest diffuse reflectance of any material or coating in the 250- to 2500-nm band. All ports of the integrating spheres had 10 mm diameters. The diameter of the sample was larger than the sample port. After the incident light was illuminated onto the samples, the diffusely reflected and transmitted light from the sample was diffused in the integrating spheres or detected through an optical fiber (CUSTOM-PATCH-2243142, Ocean Optics) connected to a spectrometer (MAYP10161, Maya2000-Pro, Ocean Optics) that is sensitive in the 400- to 1100-nm wavelength range. The integration time for the measurements was 500 ms; $R_{d}$ and $T_{t}$ were measured at five spots in the samples and averaged. A Spectralon diffuse reflectance standard (SRS-99-010, Labsphere) or a beam trap (BT610, Thorlabs) was used to measure the background of the diffuse reflectance spectrum. The double integrating sphere spectrometric system was calibrated using a Spectralon diffuse reflectance standard (SRS-20-010, Labsphere) and a transmittance filter (JCRM130, Japan Quality Assurance Organization, Japan). The $R_{d}$ difference between the measured and calibrated values was found to be within 0.7% for wavelengths below 1000 nm and within 2.5% above 1000 nm. The larger difference above 1000 nm is caused by wavelength dependencies of the integrating spheres and the spectrometer. The reflectance of the integrating spheres and the detection sensitivity of the spectrometer decrease in the near-infrared wavelengths. The difference in the $T_{t}$ measured was within 0.2%. The measurements discussed in this paper were all carried out at room temperature (23°C).

### 2.3 Data Processing Technique

The $\mu_{a}$ and $\mu_{s}^{\prime }$ were determined from the measured diffuse reflectance $R_{d,\exp}$ and total transmittance $T_{t,\exp}$. using the iMC technique.^17^ First, possible ranges of $\mu_{a}$ and $\mu_{s}^{\prime }$ values were estimated according to Ref. 22, and sets of $\mu_{a}$ and $\mu_{s}^{\prime }$ in $0.1\;{\mathrm{mm}}^{-1}$ increments were prepared. The diffuse reflectance $R_{d,\mathrm{cal}}$. and total transmittance $T_{t,\mathrm{cal}}$. were then calculated with the prepared sets of $\mu_{a}$ and $\mu_{s}^{\prime }$ and the sample geometry using a Monte Carlo code named MCML developed by Wang et al.^23^ The calculated values of $R_{d,\mathrm{cal}}$ and $T_{t,\mathrm{cal}}$ were next compared with the measured values of $R_{d,\exp}$. and $T_{t,\exp}$., and the differences between $R_{d,\mathrm{cal}}$ and $R_{d,\exp}$ and between $T_{t,\mathrm{cal}}$ and $T_{t,\exp}$ were calculated. Finally, the estimated $\mu_{a}$ and $\mu_{s}^{\prime }$ were accepted as the $\mu_{a}$ and $\mu_{s}^{\prime }$ of the samples if the relative differences were smaller than 0.5%. Otherwise, sets of $\mu_{a}$ and $\mu_{s}^{\prime }$ that minimized the differences between the calculated and measured values were taken, and the above steps were iterated repeatedly in smaller increments until the measured and estimated $R_{d}$ and $T_{t}$ agreed within the specified tolerance of 0.5%. This iterative process produced the set of epidermis, dermis, and subcutaneous fat $\mu_{a}$ and $\mu_{s}^{\prime }$ that most closely matched the measured values of $R_{d}$ and $T_{t}$. The anisotropy factor g was assumed to be 0.9, which is a typical value in many tissues.^24^ The refractive indices of epidermis, dermis, and subcutaneous fat used for this calculation were fixed at 1.34, 1.39, and 1.44 for 633 nm irradiation, respectively.^7^

### 2.4 Geometry of the Modeled Skin Tissue for Optical Penetration Depth and Energy Deposition Calculation

**Fig. 1:**
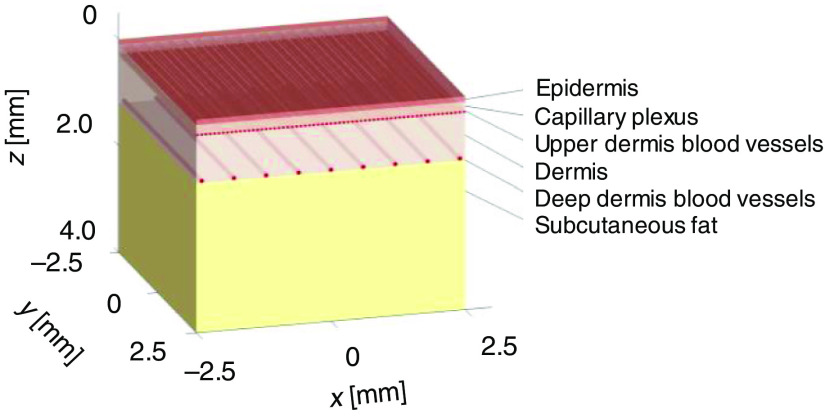
3-D structure of numerical human skin tissue model consisting of epidermis, dermis, subcutaneous fat, and three kinds of blood vessels (capillary plexus, upper dermis blood vessels, and deep dermis blood vessels).

To calculate the optical penetration depth δ and energy deposition S using the experimentally obtained absorption and reduced scattering coefficients of epidermis, dermis, and subcutaneous fat, a three-dimensional (3-D) numerical model of human skin tissue was constructed. The skin tissue model consisted of three layers, namely, the epidermis, dermis, and subcutaneous fat, as shown in Fig. 1. In the dermis layer, three different sizes of blood vessels, namely, capillary plexus, upper dermis blood vessels, and deep dermis blood vessels, were modeled to simulate the distribution of blood vessels. The parameters of the blood vessels used in the model are shown in Table 1.^25^ The capillary plexus was 150  μm in thickness. The upper dermis blood vessels and the deep dermis blood vessels lined at a depth of 340 and 1210  μm, respectively. The skin tissue model was constructed using 500×500×400 cubicle voxels. The size of each voxel was $10\times 10\times 10\;\mu m^{3}$. The depth direction of the model was set as the z axis. The skin surface was set at z=0  mm. The center of the model on the xy plane was set at (x,y)=(0  mm,0  mm). The thickness of the layers of epidermis and dermis were set to the averages of the measured values in this paper.

**Table 1:** Parameters of blood vessels.

	Center depth (μm)	Volume fraction (%)	Diameter (μm)
Capillary plexus	230	4	10
Upper dermis blood vessels	340	30	50
Deep dermis blood vessels	1210	10	80

### 2.5 Light Propagation Calculation in Human Skin Tissue

A 3-D Monte Carlo code named mcxyz developed by Jacques was adopted for calculating δ and S.^26^ This model is a computer simulation of the light distribution within a complex tissue that consists of many different types of tissues, each with its own optical properties. Uniform pencil beams with 2, 3, and 4 mm diameters were assumed to enter the numerical model vertically at (x,y)=(0  mm,0  mm). The δ and S were calculated at the wavelengths of 405, 532, 595, 632, 694, 755, 800, 980, and 1064 nm that are currently used for laser skin treatments in dermatology and plastic surgery. To calculate the light distribution in the layered skin model, the measured $\mu_{a}$ and $\mu_{s}^{\prime }$ of Asian epidermis, dermis, and subcutaneous fat at each wavelength were assigned in the numerical skin model. The absorption and reduced scattering coefficients of human blood at each wavelength are summarized in Table 2.^27^^–^^29^ The g was assumed to be 0.9.^24^ The simulation was carried out for 180 million photons to achieve sufficient distribution of light fluence in the skin tissue.

**Table 2:** Absorption coefficient $\mu_{a}$ and reduced scattering coefficient $\mu_{s}^{\prime }$ of human whole blood. The blood oxygen saturation was 96%.

Wavelength (nm)	$\mu_{a}$ (${\mathrm{mm}}^{-1}$)	$\mu_{s}^{\prime }$ (${\mathrm{mm}}^{-1}$)
405	176.03	2.53
532	23.43	2.11
595	3.89	1.96
632	0.39	1.88
694	0.19	1.77
755	0.32	1.68
800	0.44	1.61
980	0.59	1.41
1064	0.30	1.34

## 3 Results

### 3.1 Absorption Coefficient and Reduced Scattering Coefficient of Asian Epidermis, Dermis, and Subcutaneous Fat Tissues

**Fig. 2:**
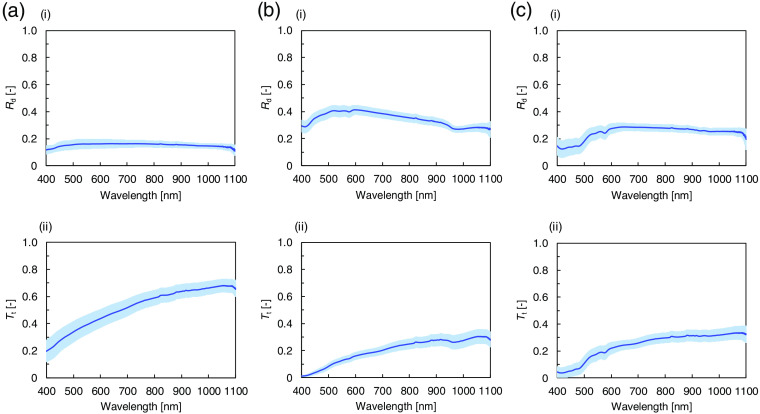
Diffuse reflectance $R_{d}$ and total transmittance $T_{t}$ spectra of Asian (a) epidermis, (b) dermis, and (c) subcutaneous fat averaged over the 15, 20, and 15 samples, respectively. The (i) upper and (ii) lower figures show $R_{d}$ and $T_{t}$, respectively. The shaded area represents the standard deviation.

**Fig. 3:**
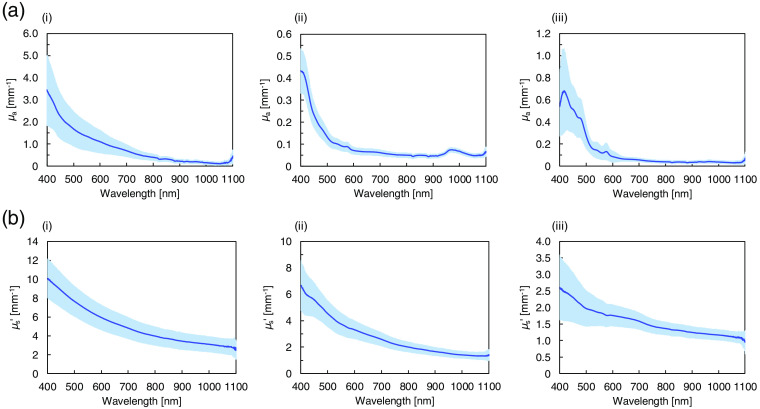
Wavelength dependences of (a) absorption coefficient $\mu_{a}$ and (b) reduced scattering coefficient $\mu_{s}^{\prime }$ of Asian (i) epidermis, (ii) dermis, and (iii) subcutaneous fat averaged over the 15, 20, and 15 samples, respectively. The shaded area represents the standard deviation.

The $\mu_{a}$ and $\mu_{s}^{\prime }$ of Asian epidermis, dermis, and subcutaneous fat were measured *in vitro* in the 400- to 1100-nm wavelength range with the double integrating sphere spectrometric system and the iMC technique. Figure 2 shows the $R_{d}$ and $T_{t}$ spectra of Asian epidermis, dermis, and subcutaneous fat measured using the double integrating sphere spectrometric system. The $\mu_{a}$ and $\mu_{s}^{\prime }$ were obtained by comparing the measured $R_{d}$ and $T_{t}$ values with the values calculated by the iMC technique. Figure 3(a) shows the $\mu_{a}$ spectra of epidermis, dermis, and subcutaneous fat. The higher standard deviations compared with the detection sensitivity of the optical setup are caused by the individual and body-site differences of the used samples. The epidermis $\mu_{a}$ decreases with increasing wavelength. The absorption in epidermis is remarkably higher than that in dermis and subcutaneous fat because of the strong effect of melanin in epidermis. Absorption by melanin increases steadily at shorter wavelengths over the broad range from 250 to 1200 nm.^30^ There is a marked dispersion of the $\mu_{a}$ values in the 400- to 600-nm wavelength range related to the difference in the melanin content between the samples obtained from photoexposed and photoprotected skin.^31^ In the dermis $\mu_{a}$ spectrum, hemoglobin absorption peaks at 403 and 573 nm and water absorption peak at 968 nm are observed. The hemoglobin absorption peaks are not pronounced because the blood in the samples was removed during the sample preparation. In the $\mu_{a}$ spectrum of subcutaneous fat, hemoglobin absorption peaks appear around 416 and 575 nm, and a broad absorption band of bilirubin appears between 400 and 500 nm. The increased standard deviation in the range of the absorption bands is caused by differences in the blood content in different tissue samples. Figure 3(b) shows the $\mu_{s}^{\prime }$ spectra of epidermis, dermis, and subcutaneous fat. The $\mu_{s}^{\prime }$ values of epidermis are noticeably higher than those of dermis and subcutaneous fat. The $\mu_{s}^{\prime }$ values decrease with increasing wavelength. The steady decrease can be attributed to the decrease in the contribution of Rayleigh scattering and the increase in the contribution of Mie scattering with increasing wavelength.^32^

### 3.2 Effect of Sample Preparation on Absorption Coefficient and Reduced Scattering Coefficient

**Fig. 4:**
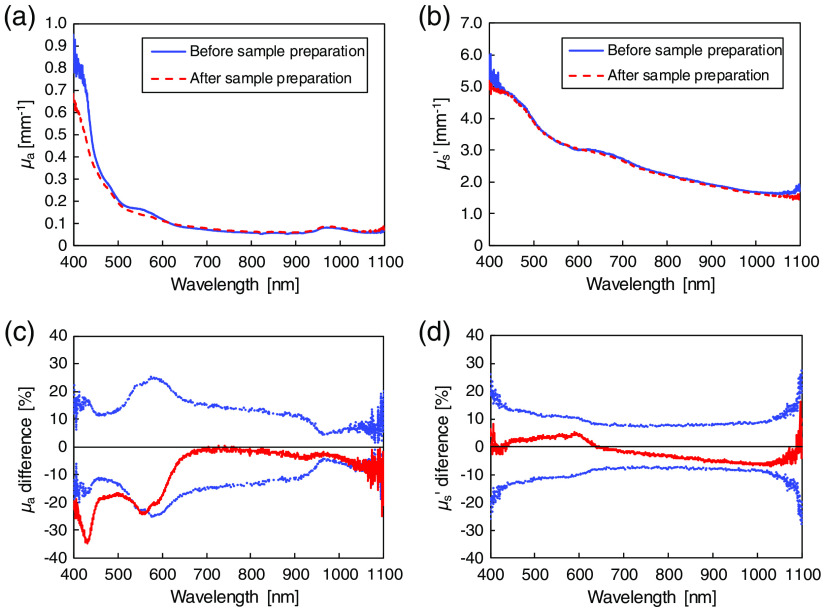
(a), (b) Wavelength dependences of $\mu_{a}$ and $\mu_{s}^{\prime }$ of epidermis + dermis before and after sample preparation. The solid and broken lines show the values before and after sample preparation, respectively. (c), (d) Spectral dependences of the relative differences between the $\mu_{a}$ and $\mu_{s}^{\prime }$ of epidermis + dermis before and after the sample preparation. The solid line and dotted lines represent the relative differences of the $\mu_{a}$ and $\mu_{s}^{\prime }$, with ±1 SD, respectively.

The sample preparation technique could have influenced the evaluation of $\mu_{a}$ and $\mu_{s}^{\prime }$.^33^^,^^34^ The effect of sample preparation during the separation of epidermis and dermis was investigated by comparing the $\mu_{a}$ and $\mu_{s}^{\prime }$ of skin tissue consisting of epidermis and dermis (epidermis + dermis) before and after the sample preparation. For the $\mu_{a}$ and $\mu_{s}^{\prime }$ measurements before the sample preparation, epidermis + dermis after the separation of subcutaneous fat was used immediately for the $R_{d}$ and $T_{t}$ measurements. For the measurements after the sample preparation, the same epidermis + dermis was used after soaking in PBS for 48 h at 4°C. Figure 4 shows the $\mu_{a}$ and $\mu_{s}^{\prime }$ spectra of epidermis + dermis before and after the sample preparation, and the relative differences of the $\mu_{a}$ and $\mu_{s}^{\prime }$ after the sample preparation. The mean $\mu_{a}$ after the sample preparation is lower than that before the sample preparation. The largest difference in the $\mu_{a}$ values is −35% at 432 nm. In the 400- to 570-nm wavelength range, the difference is significant because the blood contained in the sample was almost completely removed by the soaking solution. In the wavelength range longer than 570 nm, however, the relative difference lies within the standard deviation of the result before the sample preparation as shown in Fig. 4(c); therefore, it was judged as insignificant. The difference in the $\mu_{s}^{\prime }$ values is approximately ±10% in the entire wavelength range and shows a gradual increase with increasing wavelength. The difference is not regarded as significant. The standard deviations of both $\mu_{a}$ and $\mu_{s}^{\prime }$ increase from 1000 to 1100 nm because of the lower detection sensitivity of the used setup in the near-infrared wavelength range.

### 3.3 Optical Penetration Depth and Energy Deposition

**Fig. 5:**
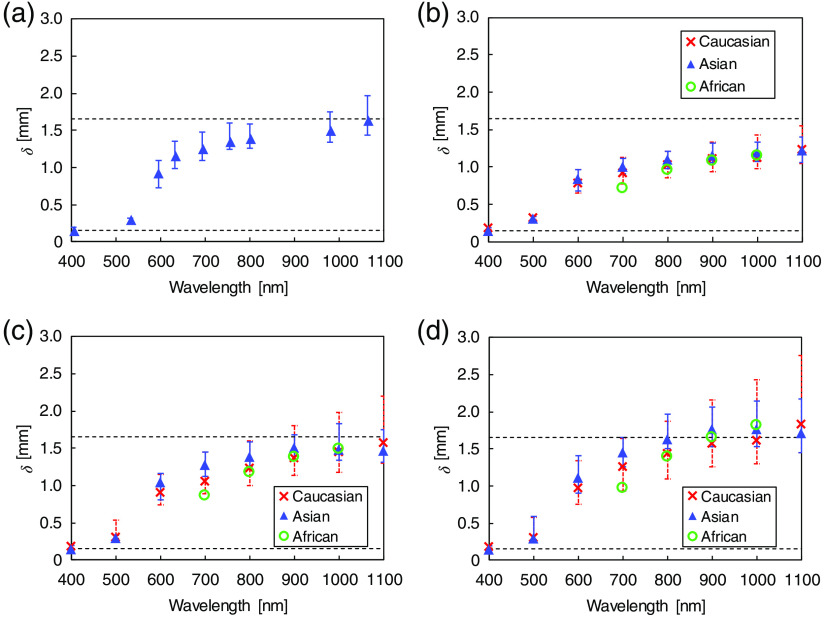
(a) Optical penetration depths δ at spot diameter of 3 mm and wavelengths currently available for clinical use in dermatology and plastic surgery. (b)–(d) Comparisons of δ at spot diameters of 2, 3, and 4 mm among the three ethnic groups. The broken lines at 0.15 and 1.65 mm show the boundaries between the epidermis and the dermis and between the dermis and the subcutaneous fat, respectively.

δ and S were calculated using the 3-D Monte Carlo simulation. The layered skin model with the measured $\mu_{a}$ and $\mu_{s}^{\prime }$ as shown in Table 3. δ was defined as the depth at which the distributed light fluence inside the skin model falls to 1/e (36.8%) of the incident fluence at the surface. Figure 5(a) shows the values of δ at the wavelengths currently available for clinical use in dermatology and plastic surgery. To estimate the dispersion of δ in skin tissue, the maximum and minimum values of δ were calculated using the standard deviations of the $\mu_{a}$ and $\mu_{s}^{\prime }$ of each tissue layer. The maximum δ was obtained from the minimum $\mu_{a}$ and $\mu_{s}^{\prime }$ (mean−1  SD), while the minimum δ was obtained from the maximum $\mu_{a}$ and $\mu_{s}^{\prime }$ (mean+1  SD). δ is dependent on the irradiation wavelength. At 405 and 532 nm, the light penetrates up to the upper part of dermis layer. At 595, 632, 694, 755, and 800 nm, the light penetrates into the deeper part of dermis layer. At 980 and 1064 nm, the light penetrates into the subcutaneous fat layer. Figures 5(b)–5(d) present the comparison of δ at the spot diameters of 2, 3, and 4 mm among the three ethnic groups. δ increases with the laser beam spot diameter because of the forward scattering in skin tissue. Thereby, lower fluence is available when a larger laser spot size is used.^35^ δ does not differ significantly among the three ethnic groups after taking into account the dispersions of $\mu_{a}$ and $\mu_{s}^{\prime }$.

**Table 3:** Absorption coefficient $\mu_{a}$ and reduced scattering coefficient $\mu_{s}^{\prime }$ (mean ± 1SD) of Asian epidermis, dermis, and subcutaneous fat at typical wavelengths available for laser skin treatments.

Wavelength (nm)	Epidermis		Dermis		Subcutaneous fat	
	$\mu_{a}$ (${\mathrm{mm}}^{-1}$)	$\mu_{s}^{\prime }$ (${\mathrm{mm}}^{-1}$)	$\mu_{a}$ (${\mathrm{mm}}^{-1}$)	$\mu_{s}^{\prime }$ (${\mathrm{mm}}^{-1}$)	$\mu_{a}$ (${\mathrm{mm}}^{-1}$)	$\mu_{s}^{\prime }$ (${\mathrm{mm}}^{-1}$)
405	3.32±1.51	9.95±2.02	0.43±0.10	6.46±1.77	0.62±0.34	2.56±0.94
532	1.44±0.69	7.04±1.48	0.10±0.03	3.96±0.89	0.15±0.07	1.89±0.45
595	1.13±0.53	6.02±1.33	0.07±0.02	3.35±0.71	0.09±0.05	1.77±0.33
632	0.94±0.41	5.55±1.25	0.07±0.02	3.06±0.62	0.07±0.03	1.72±0.30
694	0.72±0.29	4.89±1.15	0.06±0.02	2.64±0.53	0.06±0.02	1.60±0.26
755	0.49±0.19	4.28±1.04	0.05±0.01	2.20±0.43	0.04±0.02	1.43±0.22
800	0.39±0.15	3.98±1.00	0.05±0.01	2.01±0.39	0.04±0.02	1.37±0.20
980	0.17±0.09	3.17±0.85	0.07±0.01	1.45±0.29	0.04±0.02	1.18±0.17
1064	0.13±0.10	2.85±0.86	0.05±0.01	1.34±0.29	0.03±0.02	1.11±0.16

**Fig. 6:**
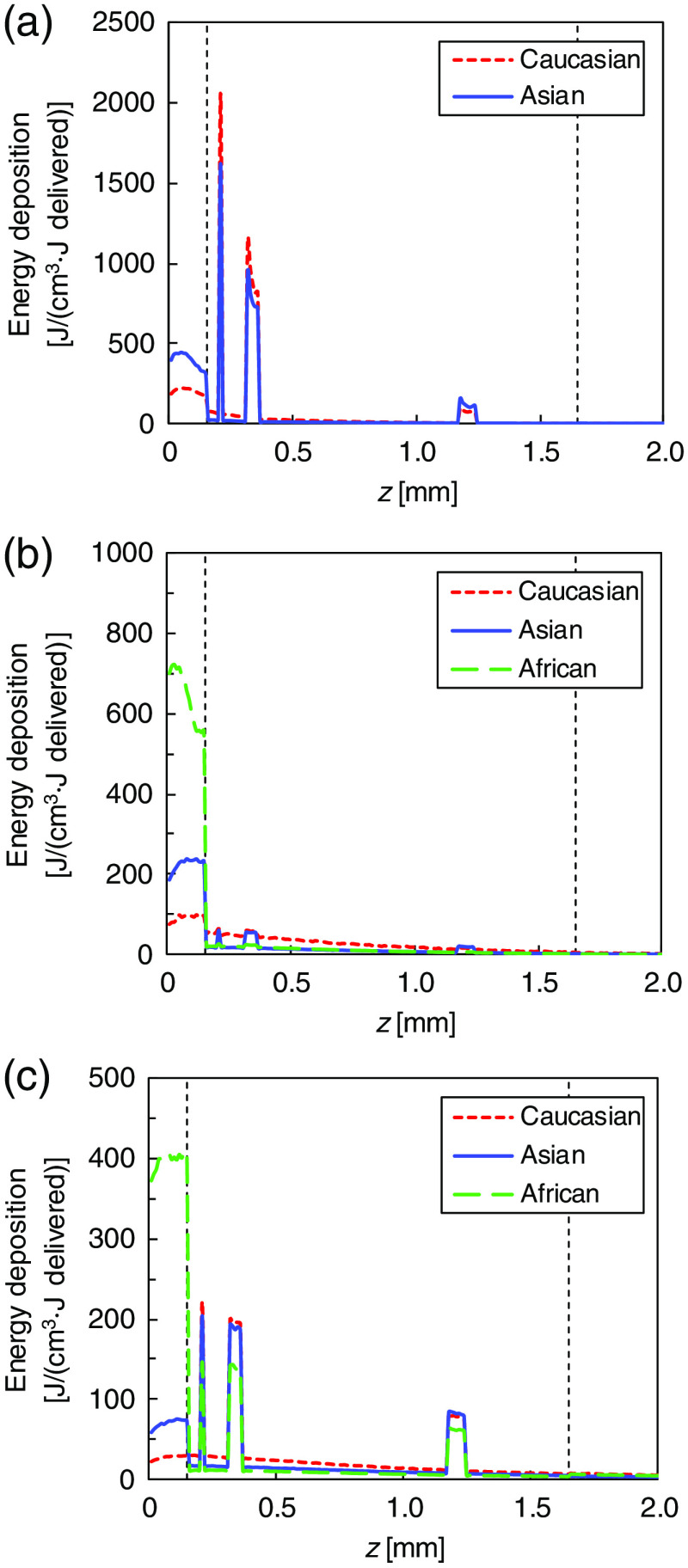
Ethnic differences in energy deposition S profiles on the depth z axis (x=y=0  mm) at the wavelengths of (a) 500, (b) 700, and (c) 900 nm. The broken lines at 0.15 and 1.65 mm show the boundaries between the epidermis and the dermis and between the dermis and the subcutaneous fat, respectively.

The S in skin tissue was obtained by multiplying the fluence and absorption coefficient of each tissue type. Figure 6 presents the ethnic differences in the S profiles along the depth z axis at 500, 700, and 900 nm. The amount of light energy delivered to each tissue is wavelength dependent. The peaks occur at the epidermis layer and the blood vessels owing to absorption by melanin pigment and hemoglobin, respectively. The energy deposited in Caucasian subcutaneous fat is larger than that in Asian and African subcutaneous fats. The maximum S at 500 nm appears at the capillary blood vessels. The maximum S in Asian skin tissue is lower than that in Caucasian skin tissue. At 700 nm, the maximum S is observed at the epidermis layer. The maximum S in Asian skin tissue is two times larger than that in Caucasian skin tissue and two times smaller than that in African skin tissue. At 900 nm, the maximum S in Caucasian and Asian skin tissues occurs at the capillary blood vessels and that in African skin tissue at the epidermis layer. The maximum S in African epidermis layer is four times larger than that at the Asian epidermis layer. S is tissue dependent and varies among the ethnic groups.

## 4 Discussion

**Fig. 7:**
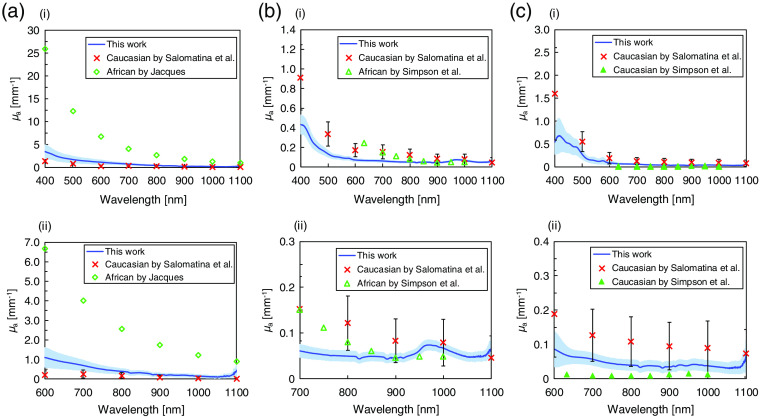
Absorption coefficient $\mu_{a}$ of Asian (a) epidermis, (b) dermis, and (c) subcutaneous fat are compared with the reported values in Caucasian and African skin in (i) the entire wavelength range and (ii) the magnified wavelength range. The crosses correspond to Caucasian epidermis, dermis, and subcutaneous fat.^10^ The open diamonds correspond to African epidermis.^14^ The open and closed triangles correspond to African dermis and Caucasian subcutaneous fat, respectively.^11^ The error bars represent the standard deviation of the reported values.

The validity of the $\mu_{a}$ and $\mu_{s}^{\prime }$ values of Asian epidermis, dermis, and subcutaneous fat was evaluated based on comparison with the reported values in Caucasian and African skin. The reported results were obtained using the same measurement system as the current work. Figure 7 shows the comparison of the $\mu_{a}$ values of each layer in Asian skin tissue with the reported results in Caucasian and African skin tissues. The $\mu_{a}$ values of Asian epidermis are higher than those of Caucasian epidermis and smaller than those of African epidermis in the entire wavelength range because darkly pigmented skin contains more melanin than lightly pigmented skin.^31^ These results show a good agreement with the *in vivo* study by Tseng et al.^13^ The $\mu_{a}$ values of Asian dermis and subcutaneous fat are comparable to the reported values^10^^,^^11^ in the wavelength range longer than 700 nm. In the shorter wavelength range, the $\mu_{a}$ values of Asian dermis are lower than the reported values. This seems to be caused by the absorption by blood. The *in vitro* $\mu_{a}$ values reported by Salomatina et al.^10^ included blood absorption. By contrast, in this paper, the effect of the blood in the dermis on the absorption was almost removed because the excisions were soaked in saline solution. In the *ex vivo* study by Simpson et al.,^11^ “dermis” included all tissue from the skin surface to the bottom of the dermis (including epidermis). Thus, the reported values included absorption by the epidermis. The $\mu_{a}$ values of Asian subcutaneous fat in the wavelength range shorter than 700 nm are lower than the $\mu_{a}$ values by Salomatina et al.^10^ owing to the blood contained in the excisions. Simpson et al.^11^ also reported the $\mu_{a}$ values, which were lower than those measured in this paper by half. They removed the blood from their samples to exclude the influence of hemoglobin on the $\mu_{a}$ values.

**Fig. 8:**
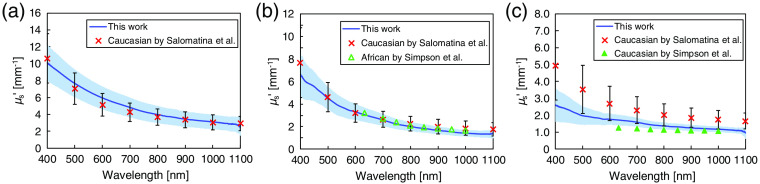
Reduced scattering coefficient $\mu_{s}^{\prime }$ of Asian (a) epidermis, (b) dermis, and (c) subcutaneous fat are compared with the reported values of Caucasian and African skin tissues. The crosses correspond to Caucasian epidermis, dermis, and subcutaneous fat.^10^ The open and closed triangles correspond to African dermis and Caucasian subcutaneous fat, respectively.^11^ The error bars represent the standard deviation of the reported values.

Figure 8 shows the comparison of the $\mu_{s}^{\prime }$ values of each layer in Asian skin tissue with the reported values in Caucasian and African skin tissues. The $\mu_{s}^{\prime }$ values of Asian epidermis and dermis are similar to the reported values^10^^,^^11^ in magnitude and slope across the entire wavelength range. This indicates that the epidermis and dermis of these groups have similar compositions in terms of the average scatter size and density. The $\mu_{s}^{\prime }$ values of Asian subcutaneous fat are lower than the data presented by Salomatina et al.^10^ They attributed the increased scattering coefficient to the presence of connective tissue septa composed of thicker and denser collagen–elastin net in the subcutaneous fat samples obtained from the facial or scalp area. Compared with the *ex vivo* study by Simpson et al.,^11^ the $\mu_{s}^{\prime }$ values are higher. This disagreement is likely related to unavoidable differences in the tissue sample storage and preparation procedures used. Their samples were refrigerated for 5 days and allowed to return to room temperature before being dissected.

**Fig. 9:**
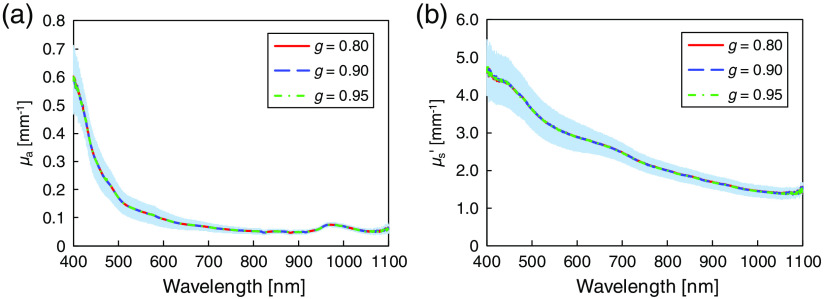
Wavelength dependences of (a) $\mu_{a}$ and (b) $\mu_{s}^{\prime }$ of epidermis + dermis for g=0.80 (red), 0.90 (blue), and 0.95 (green). The shaded area represents the standard deviation for g=0.90.

In the iMC technique, the g value was assumed to be 0.9 for each layer of skin tissue. So far, the measured $\mu_{a}$ and $\mu_{s}^{\prime }$ values of each layer of human skin tissue have the insensitivity to g used for optical property analysis based on the iMC technique.^11^ A small (<1%) variation was observed in obtained $\mu_{s}^{\prime }$ values and a negligible change was observed in $\mu_{a}$ values when g values were changed from 0.8 to 0.95. Figure 9 shows the $\mu_{a}$ and $\mu_{s}^{\prime }$ values of the samples after the sample preparation measured in Sec. 3.2 using g=0.8, 0.9, and 0.95. The $\mu_{a}$ and $\mu_{s}^{\prime }$ values obtained using the different g values are almost same, which indicates that the measurement of $\mu_{a}$ and $\mu_{s}^{\prime }$ values is insensitive to the value of g used in the iMC technique. The standard deviations of the $\mu_{a}$ and $\mu_{s}^{\prime }$ derived from the individual and body-site differences of the used samples are more dominant than the variations in obtained $\mu_{a}$ (<2%) and $\mu_{s}^{\prime }$ (<1%) caused by changing g values, respectively.

The effect of the sample preparation on the $\mu_{a}$ values in the wavelength range of the hemoglobin absorption band was significant. The volume fraction of blood lost by the sample preparation was estimated to be 0.2% from the comparison of the $\mu_{a}$ values before and after the sample preparation. Our results are inconsistent with the reported value of 5.4%,^25^ which indicates that the absence of blood was mainly caused by the immersion in saline solution during the delivery of the specimens. Roggan et al. postulated that the short-time storage of specimens in saline solution caused the loss of hemoglobin, resulting in considerable changes to the $\mu_{a}$ values.^33^ The measured $\mu_{a}$ values were therefore regarded as bloodless parameters. To apply such parameters for clinical purposes, the $\mu_{a}$ values of blood need to be considered to reproduce the actual *in vivo* situation.

The optical penetration depth and energy deposition in skin tissue have important clinical implications because of their effect on the treatment area and the choice of irradiation parameters. Several clinical and theoretical studies associated with photodynamic therapy (PDT) have demonstrated that the optical penetration depth is essential for determining the light dose that generates photodynamic action to establish effective PDT dosimetries.^36^^–^^38^ Our calculation indicates that the differences in δ are not significant among the ethnic groups. This result suggests that, under the assumption that the distribution of photosensitizer prodrug administrated in skin tissue is constant among the three ethnic groups, the differences among the skin types have insignificant effects on the PDT treatment depth. In the S calculation, the degree of the peaks observed at the epidermis and the blood vessels are wavelength-dependent and have significant differences among the ethnic groups. The S of the epidermis is affected by the difference in the $\mu_{a}$ values of epidermis among the skin types. The S distribution at the wavelengths of interest is regarded as a heat source in the skin tissue heat transport analysis for quantifying the thermal skin damage.^39^ The ethnic difference in the damage to the epidermis and blood vessels is thus expected to affect treatment outcomes. Such analysis leads to the effective design and clinical application of accurate irradiation parameters to minimize complications such as burning, dyspigmentation, and scarring induced by laser skin treatments of pigmented lesions and vascular lesions.^40^

The measured skin specimens were obtained from a variety of body sites, ages, and sexes. This may have introduced variations to the $\mu_{a}$ and $\mu_{s}^{\prime }$ spectra. The accumulation of sufficient knowledge of these differences will allow for the construction of virtual human skin models for various sites. Using these skin models in combination with light propagation calculations will improve the precision of the produced light distributions and the computational analysis of light–tissue interactions for laser skin therapy.

## 5 Conclusion

The $\mu_{a}$ and $\mu_{s}^{\prime }$ of Asian epidermis, dermis, and subcutaneous fat were experimentally measured in the 400- to 1100-nm wavelength range using the double integrating sphere spectrometric system and the iMC technique. The validity of the measured values was confirmed through comparison with the reported results for Caucasian and African skin tissues. Using a layered skin model, the measured $\mu_{a}$ and $\mu_{s}^{\prime }$, and δ and S were calculated. The $\mu_{a}$ values of Asian epidermis differ prominently from those of Caucasian and African epidermises, while the $\mu_{s}^{\prime }$ values of each layer in Asian skin tissue are similar to those of the other ethnic skin tissues. The analysis revealed that the ethnic differences in the $\mu_{a}$ and $\mu_{s}^{\prime }$ have little effect on the PDT treatment depth, while the differences present in S among Asian, Caucasian, and African epidermises can cause large variances in the heat production in skin tissue. The experimentally measured $\mu_{a}$ and $\mu_{s}^{\prime }$ values of Asian human epidermis, dermis, and subcutaneous fat allow for the analysis of light propagation in Asian skin tissues for evaluating the efficacy and safety of laser therapy for Asian skin diseases.
