# Measurement of absorption and reduced scattering coefficients in Asian human epidermis, dermis, and subcutaneous fat tissues in the 400- to 1100-nm wavelength range for optical penetration depth and energy deposition analysis (Errata)

**DOI:** 10.1117/1.JBO.26.1.019802

**Published:** 2021-01-21

**Authors:** Yu Shimojo, Takahiro Nishimura, Hisanao Hazama, Toshiyuki Ozawa, Kunio Awazu

**Affiliations:** a Osaka University, Graduate School of Engineering, Suita, Japan; b Osaka City University, Graduate School of Medicine, Department of Dermatology, Osaka, Japan; c Osaka University, Graduate School of Frontier Biosciences, Suita, Japan; d Osaka University, Global Center for Medical Engineering and Informatics, Suita, Japan

## Abstract

The errata correct errors that appeared in Table 2 of the published article.

This article [J. Biomed. Opt. 25(4), 045002 (2020), doi: 10.1117/1.JBO.25.4.045002] was originally published on 30 April 2020 with errors in Table 2. The legend did not mention blood oxygen saturation, and the third column showed incorrect values for reduced scattering coefficient ${\mu_{s}}^{\prime }$ (${\mathrm{mm}}^{-1}$) of human whole blood at wavelengths of 405, 532, 595, 632, 694, 755, 800, 980, and 1064 nm. The blood oxygen saturation is 96%, and the correct values for ${\mu_{s}}^{\prime }$ at each wavelength are 2.53, 2.11, 1.96, 1.88, 1.77, 1.68, 1.61, 1.41, and $1.34\;{\mathrm{mm}}^{-1}$, respectively. These errors did not affect the results presented in this article. The corrected table is reproduced below:

**Table 2:** Absorption coefficient $\mu_{a}$ and reduced scattering coefficient ${\mu_{s}}^{\prime }$ of human whole blood. The blood oxygen saturation was 96%.

Wavelength (nm)	$\mu_{a}$ (${\mathrm{mm}}^{-1}$)	${\mu_{s}}^{\prime }$ (${\mathrm{mm}}^{-1}$)
405	176.03	2.53
532	23.43	2.11
595	3.89	1.96
632	0.39	1.88
694	0.19	1.77
755	0.32	1.68
800	0.44	1.61
980	0.59	1.41
1064	0.30	1.34

The errors in the article were corrected on 19 January 2021.

